# Affinity Purification and Comparative Biosensor Analysis of Citrulline-Peptide-Specific Antibodies in Rheumatoid Arthritis

**DOI:** 10.3390/ijms19010326

**Published:** 2018-01-22

**Authors:** Eszter Szarka, Petra Aradi, Krisztina Huber, Judit Pozsgay, Lili Végh, Anna Magyar, Gergő Gyulai, György Nagy, Bernadette Rojkovich, Éva Kiss, Ferenc Hudecz, Gabriella Sármay

**Affiliations:** 1 Department of Immunology, Eötvös Loránd University, 1117 Budapest, Hungary; szarka.eszter@ttk.elte.hu (E.S.); aradi.petra@gmail.com (P.A.); krisztina.huber@gmail.com (K.H.); pozsgayj@gmail.com (J.P.); lilivegh92@gmail.com (L.V.); 2 MTA-ELTE Research Group of Peptide Chemistry, Hungarian Academy of Science, Eötvös Loránd University, 1117 Budapest, Hungary; magyar@elte.hu (A.M.); fhudecz@caesar.elte.hu (F.H.); 3 Laboratory of Interfaces and Nanostructures, Institute of Chemistry, Eötvös Loránd University, 1117 Budapest, Hungary; gyulaigor@gmail.com (G.G.); kisseva@chem.elte.hu (É.K.); 4 Department of Rheumatology, 3rd Department of Internal Medicine, Semmelweis University, 1125 Budapest, Hungary; gyorgyngy@gmail.com; 5 Rheumatology, Buda Hospital of the Hospitaller Order of St. John of God, 1023 Budapest, Hungary; rojkovich.b@gmail.com

**Keywords:** rheumatoid arthritis, citrulline-peptides, autoantibody, affinity, cross-reaction, targeting

## Abstract

Background: In rheumatoid arthritis (RA), anti-citrullinated protein/peptide antibodies (ACPAs) are responsible for disease onset and progression, however, our knowledge is limited on ligand binding affinities of autoantibodies with different citrulline-peptide specificity. Methods: Citrulline-peptide-specific ACPA IgGs were affinity purified and tested by ELISA. Binding affinities of ACPA IgGs and serum antibodies were compared by surface plasmon resonance (SPR) analysis. Bifunctional nanoparticles harboring a multi-epitope citrulline-peptide and a complement-activating peptide were used to induce selective depletion of ACPA-producing B cells. Results: *K*_D_ values of affinity-purified ACPA IgGs varied between 10^−6^ and 10^−8^ M and inversely correlated with disease activity. Based on their cross-reaction with citrulline-peptides, we designed a novel multi-epitope peptide, containing Cit-Gly and Ala-Cit motifs in two–two copies, separated with a short, neutral spacer. This peptide detected antibodies in RA sera with 66% sensitivity and 98% specificity in ELISA and was recognized by 90% of RA sera, while none of the healthy samples in SPR. When coupled to nanoparticles, the multi-epitope peptide specifically targeted and depleted ACPA-producing B cells ex vivo. Conclusions: The unique multi-epitope peptide designed based on ACPA cross-reactivity might be suitable to develop better diagnostics and novel therapies for RA.

## 1. Introduction

Anti-citrullinated protein/peptide antibodies (ACPA) are highly specific and sensitive clinical markers of rheumatoid arthritis (RA), a chronic inflammatory autoimmune disease [1]. Approximately 70–80% of RA patients are ACPA positive, and high levels of ACPA have been associated with bad prognosis of the disease [2]. ACPA is produced against citrullinated self-proteins, emerging as a result of the activation of peptidyl arginine deiminase (PAD) enzymes [3,4]. PADs are activated at inflammatory sites and in the presence of a high level of intracellular free calcium in activated cells. PADs deiminate arginine to citrulline and this post-transcriptional modulation drastically modifies the structure of the protein, initiating unfolding [5]. The newly appearing citrullinated epitopes will then be recognized by the immune system, resulting in the production of ACPA [6].

Most of the citrullinated target molecules of ACPA are localized in the joints, such as collagen II [7], fibrinogen/fibrin [8,9], and the intracellular protein, vimentin [10,11], which is released from macrophages in the inflamed synovia [12]. Binding of ACPA to synovial target proteins in vitro and in vivo has been demonstrated [13]. Deposition of ACPA and ACPA containing immune complexes induce the release of inflammatory cytokines, such as TNFα, eventually leading to the escalation of inflammation [8,14,15]. ACPA appears many years before the disease onset in the sera of patients, and its detection is extremely important for early diagnosis. ACPA is not only a specific and sensitive disease marker, but also plays key role in the pathogenicity of RA [16].

A wide variety of citrulline containing peptides (Cit-peptides) are recognized by ACPA, including peptides representing citrullinated sequences in the major proteins of the joints, such as fibrinogen [8] and type II collagen [7]; the intracellular structural protein, vimentin, which is also detected in the synovium [11,17,18]; and peptides derived from bacterial (α-enolase) [19] and viral (Epstein–Barr virus nuclear antigen (EBNA)) origin [20]. An ACPA cross-reacting, citrullinated peptide, derived from filaggrin produced by epithelial cells, has also been described [9,21] and used in the first diagnostic tests of RA.

Citrullination in general is a physiological process; it may occur for example upon gene regulation [22], apoptosis [23], inflammation [24] and neutrophil extracellular trap (NET) formation [25] without inducing an autoimmune response. In RA, however, probably due to the abnormal humoral immune response, citrullinated proteins induce ACPA production in the vast majority of patients. Since ACPA is a crucial player and has a pathological role in RA, detailed characterization of this autoantibody would be of great importance both for diagnostic accuracy and prognostic evaluation.

Many groups described the multiple specificity and cross-reactivity of ACPA [26,27,28,29,30], and several citrullinated peptide epitopes have been identified, however, only limited data are available so far on the binding affinity of individual purified ACPA IgGs. Therefore, our aim was to analyze in parallel by Surface Plasmon Resonance (SPR) and ELISA both the affinity and the specificity of affinity purified autoantibodies from RA patients.

In accordance with previous data, we detected a high degree of cross-reactivity between affinity purified ACPA IgGs, with apparent dissociation constants (*K*_D_) between 10^−6^ and 10^−8^ M. Based on the pattern of cross-reaction, we designed a novel multi-epitope peptide that was specifically recognized by RA sera and was able to deplete specific B cells from in vitro culture. Thus, we suppose that this multi-epitope peptide might be further developed for RA diagnosis and therapy.

## 2. Results

### 2.1. Affinity Purification of ACPA

We have synthesized a panel of Cit-peptides corresponding to known ACPA epitopes, listed in Table 1, and prepared as insoluble matrixes for the immune-purification of ACPA from the sera of selected RA patients. Patients’ selection was based on the positivity of the sera in an in house Cit-peptide-based ELISA [31,32].

**Figure 1:**
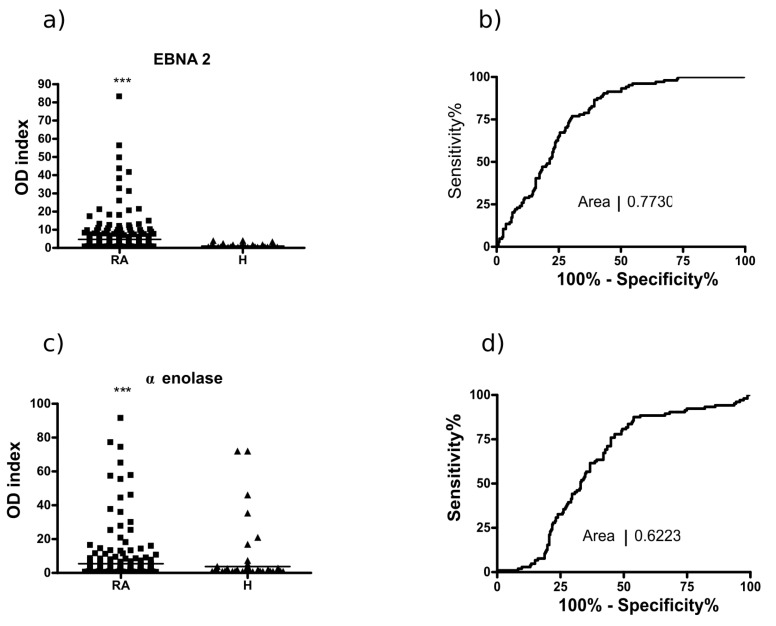
Recognition of citrulline-containing EBNA-2 and α-enolase peptide epitopes by sera of patients (RA, *n* = 177) and healthy volunteers (H, *n* = 104): (**a**,**c**) OD index: OD with Cit-peptide/OD with Arg peptide; and (**b**,**d**) receiver operating (ROC) curves. *** *p* ≤ 0.001. RA: rheumatoid arthritis.

The citrulline-containing filaggrin19, collagen II, fibrin β60–74 and vimentin peptides were already thoroughly tested with our cohort of serum samples [31,32,37]. Therefore, we only show here the screening of sera of RA patients (*n* = 177) and healthy volunteers (*n* = 104) with arginine- and citrulline-containing EBNA-2 and α-enolase peptides, respectively (Figure 1).

EBNA-2 and α-enolase peptides detected autoantibodies in RA serum samples by 48% and 27% sensitivity, respectively. For comparison, the sensitivity values for the previously tested Cit-filaggrin, fibrin, collagen II and vimentin peptides were 62%, 56%, 44% and 41%, respectively [31,32]. Based on these data, we have selected the sera, which had a high OD index at least with one of the peptides and purified the anti-peptide antibodies by affinity chromatography. ACPAs were purified in two steps [38]. First the IgG fractions were obtained on a protein G column and this was followed by the affinity purification of anti-peptide antibodies on the corresponding citrulline containing filaggrin19-, collagen II-, fibrin-, vimentin- and α-enolase-peptide-coated matrixes, respectively.

### 2.2. Cross-Reaction of Affinity-Purified ACPA IgG Fractions

**Figure 2:**
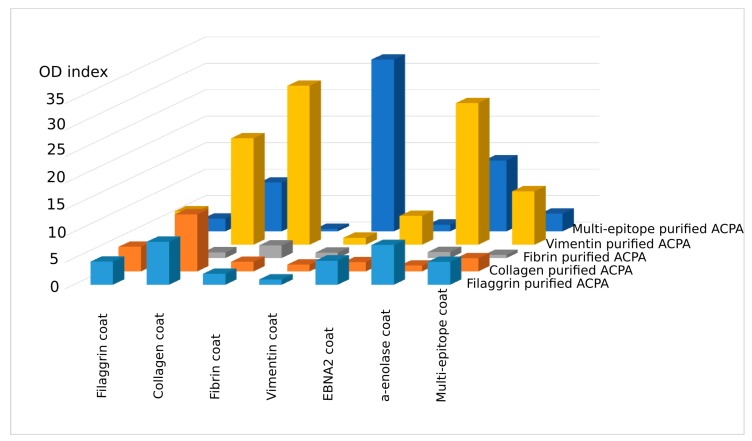
Reactivity of affinity-purified IgG fractions with the relevant and irrelevant citrulline-containing peptides. OD ratios that are OD with Cit/OD with Arg-containing peptides are shown. Cut-off values for each Cit- and Arg-containing peptide pair were calculated from OD indexes of 120 healthy samples (the means of OD indexes + 2 × SD). These were below 1.5 for all peptides. Results of a typical experiment.

To see if the affinity-purified anti-peptide antibodies were able to recognize a different peptide, ELISA plates were coated with the individual peptides directly (fibrin, enolase, and EBNA-2) or indirectly, using neutravidin-coated plates and biotinylated peptides (filaggrin, collagen II, or vimentin) and the binding of the affinity-purified IgG fractions was monitored (Figure 2).

Interestingly, the affinity-purified IgG prepared on Cit-filaggrin19 peptide recognized citrulline-containing collagen II, fibrin β, EBNA-2 and α-enolase as well, besides the filaggrin19; IgG prepared on Cit-collagen II peptide bound to citrulline-containing filaggrin, fibrin β, and EBNA-2 peptides, while IgG purified on Cit-vimentin peptide recognized all Cit-peptides tested, although at different levels. ACPA purified on the Cit-fibrin β peptide showed the lowest degree of cross-reactivity in ELISA.

We compared the Cit-peptide sequences and observed that certain short motifs containing Ala-Cit and/or Cit-Gly residues are present in all peptides. We supposed that both short motifs might be important for the recognition. Thus, we designed and synthesized a novel “multi-epitope” peptide consisting of two-two copies of Ala-Cit and Cit-Gly motifs separated with a neutral spacer, SGSG. As expected, IgG purified on citrulline-containing multi-epitope peptide-coated matrix recognized all other peptides, but with different intensity (Figure 2, dark blue columns). Conversely, the multi-epitope peptide bound to almost all other ACPA IgG, fibrin β was the exception. These data verify earlier data suggesting that ACPAs are highly cross-reactive and indicate that Ala-Cit as well as Cit-Gly motifs have importance in recognition.

### 2.3. Analysis of the Multi-Epitope Peptide by ELISA Screening of RA Sera

Next, we screened 210 RA sera and 90 healthy control samples using the biotinylated, citrulline- or arginine-containing multi-epitope peptide as a coat in ELISA.

**Figure 3:**
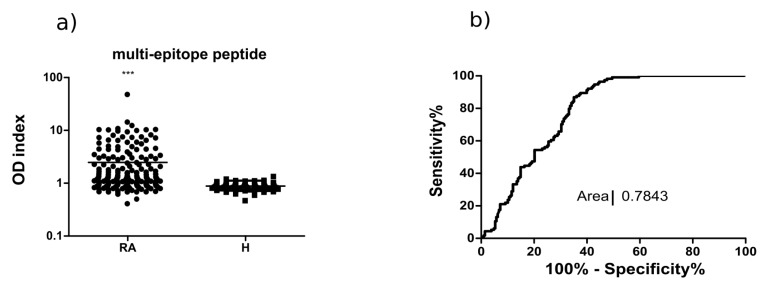
The Cit-multi-epitope peptide identifies RA sera with the highest specificity and 66% sensitivity: (**a**), ELISA, OD indexes (OD with Cit-peptide/OD with Arg-peptid) of RA (*n* = 210) and healthy (*n* = 90) samples, *** *p* ≤ 0.001; and (**b**) ROC curve of ELISA. Area under the curve (AUC): 0.7843.

The designed multi-epitope peptide identified ACPA in RA serum samples with 66% sensitivity, while none of the healthy control sera showed binding (Figure 3). This sensitivity value is somewhat higher than that of fibrin β peptide, and the shape of the ROC curve (AUC 0.7843) suggests that a diagnostic test based on the multi-epitope peptide would be more accurate.

### 2.4. SPR Analysis of Affinity-Purified Antibodies and Serum Antibodies of RA Patients

For affinity measurements of IgGs purified from RA sera on insolubilized citrulline-containing filaggrin19, collagen II, fibrin β, vimentin and multi-epitope peptides, we first had to test the ability of the individual peptides to immobilize on GLH sensor chip. An amine coupling immobilization strategy was used. Immobilization buffers were selected separately for each peptide, according to their isoelectric point. From these five peptides, only filaggrin19, vimentin and the multi-epitope peptide could couple covalently to the surface of the GLH chip. Therefore, fibrin β and collagen II peptides were biotinylated and immobilized using the neutravidin-coated NLC chip.

First, affinity-purified IgG fractions of anti-peptide antibodies were tested on various immobilized Cit-peptides. The apparent equilibrium dissociation constants (*K*_D_) were in the micromolar–10 nanomolar range, and, in the ELISAs, a high level of cross-reactivity could be observed (Table 2). These affinity-purified IgG fractions might be dominated by the product of single clone but also may contain IgG secreted by multiple B cell clones with different affinities. Therefore, the *K*_D_ values measured are corresponding to an average apparent *K*_D_.

#### 2.4.1. *K*_D_ Determination of Serum Antibodies from RA Patients Using Immobilized Cit-Peptide on GLH Chip

**Figure 4:**
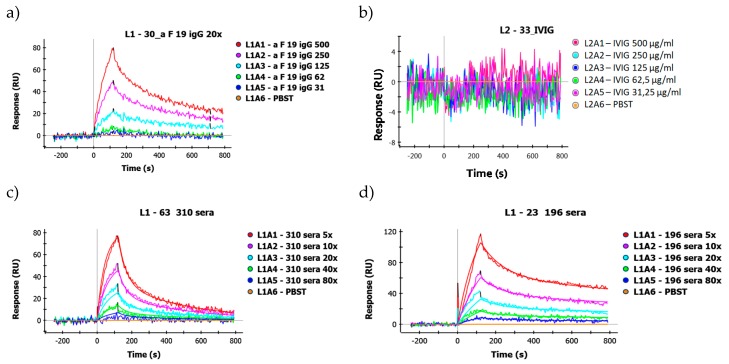
Representative sensograms of: (**a**) Cit-filaggrin19-affinity purified IgG; (**b**) negative control IVIG; and two typical sera with: (**c**) higher; and (**d**) lower affinity on Cit-filaggrin19 coupled GLH chip.

Figure 4a shows representative sensograms of IgG purified by affinity chromatography from the serum of an RA patient on immobilized Cit-filaggrin19 peptide. As a negative control, intravenous immunoglobulin (IVIG) was used, pooled from the plasma of more than a thousand healthy blood donors, which clearly did not show any specific binding (Figure 4b).

To calculate the *K*_D_ values for serum samples, the concentration of an individual peptide specific IgG was estimated in all sera by ELISA, using a standard concentration series from the given affinity purified IgG fraction. Typical binding curves of two serum samples are shown on Figure 4c,d.

**Figure 5:**
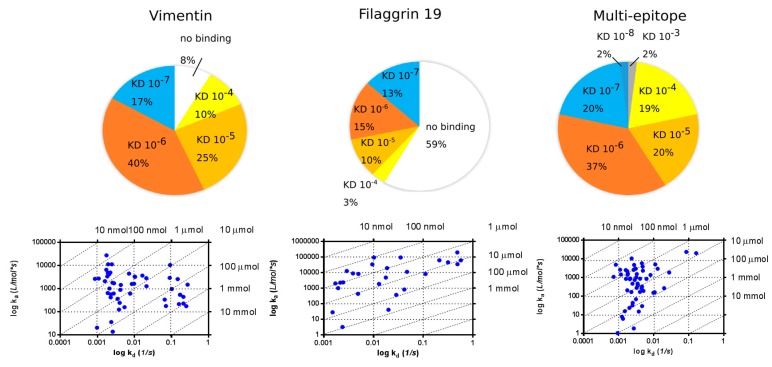
Distribution of apparent *K*_D_ values of serum antibodies as measured from RA samples on GLH chip (**top**); and isoaffinity diagrams (**bottom**).

The distribution of the apparent *K*_D_ values of serum antibodies measured on Cit-vimentin, Cit-filaggrin19 and multi-epitope peptides immobilized by covalent coupling is shown in Figure 5. The majority of apparent *K*_D_ values were in the range 10^−4^–10^−7^ M. All sera selected for this assay (*n* = 68) were highly positive for the given Cit-peptide in ELISA. SPR analysis had shown that 92% of sera bound to Cit-vimentin and all samples bound to the multi-epitope peptide on the chip. The distribution of *K*_D_ values was similar; however, only 41% of the Cit-filaggrin19 peptide-positive sera bound to this peptide under the flow condition on the chip. The association rate constant (k_a_)/dissociation rate constatnt (k_d_) plots (isoaffinity diagram) shown at the lower panels of the figure represent the data of individual sera. In general, more sera have shown a lower dissociation rate from the multi-epitope peptide as compared to the other two.

#### 2.4.2. Binding of Serum IgG to the Biotinylated Peptides Immobilized on Neutravidin-Coated Chip (NLC)

**Figure 6:**
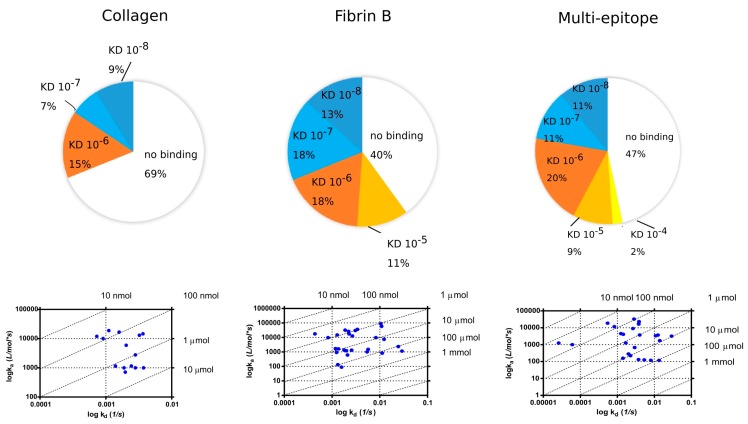
Distribution of apparent *K*_D_ values measured from RA sera on biotinylated Cit-collagen II, Cit-fibrin β and multi-epitope peptides immobilized on NLC chip.

Fibrin and collagen II peptides that did not immobilize on GLH chip were biotinylated and immobilized on a neutravidin-coated surface. Additionally, multi-epitope peptide was also biotinylated and re-tested on NLC chip (Figure 6).

When the biotinylated peptides were immobilized on the NLC chip, 31% of selected RA sera bound to Cit-collagen peptide with apparent *K*_D_ of 10^−6^–10^−8^ M, while 60% of sera bound to the Cit-fibrin β peptide with 10^−5^–10^−8^ M. Interestingly, although a lower proportion of sera (53%) showed binding to the multi-epitope peptide immobilized on the NLC chip, among those which bound, a larger proportion showed higher apparent *K*_D_ (10^−8^ M).

**Figure 7:**
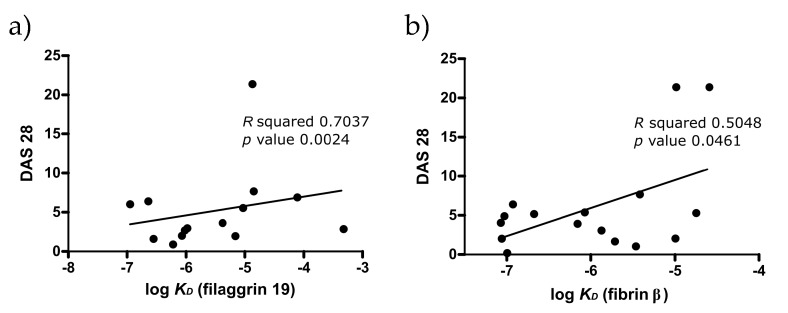
Correlation between the disease severity (DAS28) and the apparent binding affinity (*K*_D_) of serum antibodies from RA patients, (**a**) measured on filaggrin 19 peptide, (**b**) on fibrin β peptide.

Low avidity ACPA was previously shown to associate with a higher rate of joint destruction [39]. Therefore, we tested if the disease activity score, DAS28, correlates with the apparent *K*_D_ (Figure 7). We found an inverse correlation between DAS and *K*_D_ of IgG purified on Cit-filaggrin19 peptide, and on Cit-fibrin β peptide, indicating that low *K*_D_ auto-antibodies are more pathogenic.

### 2.5. Selective Depletion of Autoantibody Producing B Cells Ex Vivo by the Multi-Epitope Peptide Covalently Coupled to Poly (Lactic-Co-Glycolic Acid) (PLGA) Nanoparticles (NP)

**Figure 8:**
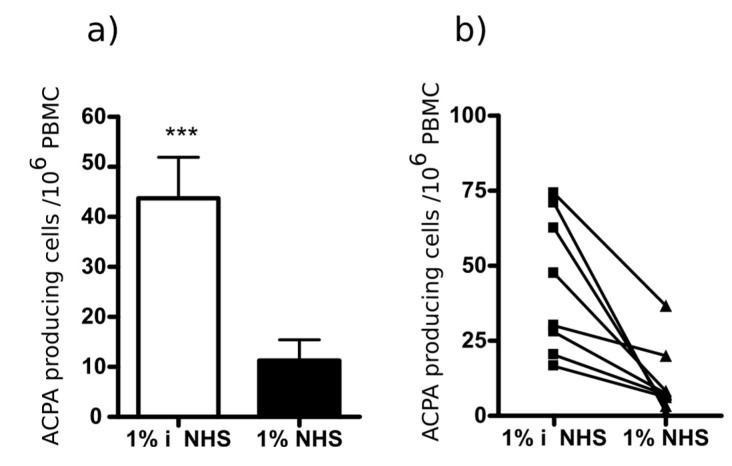
Ex vivo inhibition of the Cit-peptide-specific antibody production by bifunctional PLGA nanoparticles covalently coupled to multi-epitope peptide and a complement-activating peptide. Antibody synthesis was tested by ELISpot assay according to a modified procedure of the Manufacturer (Mabtech, Stockholm, Sweden). As complement source, 1% normal human sera (NHS) was used; for the negative control, the sera were heat-inactivated (iNHS). (**a**) Average number of peptide-specific antibody-secreting cells (ELISpots) from 10^6^ PBMC of eight RA patients; *** *p* ≤ 0.001; and (**b**) ELISpot data of individual RA patients.

We have reported earlier that bifunctional nanoparticles covalently coupled to Cit-fibrinβ60-74 peptide and a peptide inducing cell lysis deplete the peptide-specific B cells in ex vivo cultures of PBMC from RA patients. Here, we tested if the newly designed multi-epitope peptide would have a similar effect. Therefore, the multi-epitope peptide and a complement activating peptide, a modified form of the peptide derived from HIV1 GP120 [32,40] (Ac-^233^C(Acm)NNQTFNGTGPC(Acm)TNV^247^-K-NH_2_, (Acm: acetamido-methyl)) were simultaneously coupled to PLGA nanoparticles, and added to pre-activated PBMC from RA patients in the presence or absence of complement source (1% normal human sera (NHS)). The Cit-peptide specific antibody production was monitored by ELISpot assay. The bifunctional PLGA nanoparticles targeted to B cells recognizing the multi-epitope peptide significantly suppressed the peptide specific IgG secretion in the presence of complement (Figure 8), while total IgG production was not affected [32].

These data indicate that the newly designed multi-epitope peptide might be suitable for the development of autoantigen-specific immunotherapy.

## 3. Discussion

In the present study, we have analyzed the specificity and the apparent affinity of anti-peptide antibodies isolated from the sera of RA patients by affinity chromatography. The citrulline-containing peptides we used for the isolation and analysis represent the immunodominant epitopes of fibrin β chain, filaggrin, collagen II, vimentin, α-enolase and EBNA-2. Most of the affinity purified IgG fractions recognized other Cit-peptides, beside the ones used for the isolation, and their apparent *K*_Ds_ were comparable. Additionally, in some cases (e.g., anti-Cit-vimentin), anti-peptide antibodies bound to a different peptide with even higher affinity, indicating a high degree of cross-reactivity. These data are in accord with previous work of Rossi et al. showing by biosensor analysis that ACPA purified on viral peptides, VCP1 and VCP2 cross-react with a histon peptide (HCP1) and bind with a higher apparent affinity to HCP1 than to VCPs [41]. Comparing the individual peptide sequences, we have observed that all peptides contain Cit-Gly and/or Ala-Cit motifs, which are seemingly essential for the recognition. Thus, we designed an artificial peptide, containing two copies of each small motif separated with a neutral spacer. This “multi-epitope” peptide was recognized by sera from RA patients with 66% sensitivity and 98% specificity, and the binding affinities were comparable with that of the natural citrulline-containing peptides. Antibodies purified on the multi-epitope peptide cross-reacted with most other peptides tested, although at different degrees. The functional activity of the multi-epitope peptide was equivalent to that of Cit-fibrin β peptide [32]; it was able to specifically deplete Cit-peptide-specific B cells in vitro, thus significantly inhibiting ACPA production.

Cross-reactivity of ACPA was reported by several groups, and the concept that Cit-Gly motif has importance in the recognition was also studied earlier [26,29,30,36,41,42]. Several combinations of Cit-peptides and ACPAs have been tested for cross-reactivity: antibodies to Cit-EBNA (35–58) strongly cross-reacted with the immunodominant epitope of citrullinated fibrin [43] and vice versa; and two proteins encoded by Epstein-Barr virus (EBV), EBNA-1 and EBNA-2 were specifically recognized by antibodies of the ACPA family [44]. Additionally, monoclonal antibodies obtained from an RA patient and specifically directed against a citrullinated fibrin peptide also recognized Cit-peptides derived from enolase and vimentin [28]. Recently, Trier et al. [42] concluded that ACPAs are a collection of largely cross-reacting but occasionally non-cross-reactive antibodies. Cross-reactivity largely depends on the Cit-Gly motif in combination with the peptide backbone, while non-cross-reactive ACPAs depend on specific citrullinated epitopes, rather than a citrulline-containing motif [42,45].

The data presented here expand these earlier findings. First, a panel of affinity purified anti-peptide antibodies were screened with a panel of Cit-peptides representing immunodominant epitopes of in vivo ACPA target proteins; and, second, in addition to binding studies by ELISA, affinities of the interaction were also analyzed by SPR. Both the specificity and the affinity play a key role in the pathological effect of an antibody, therefore we have monitored these two parameters by analyzing their interactions with seven Cit-peptides derived from major target proteins of ACPA.

The suggested central role of Cit-Gly [42,45] in the recognition of autoantigens by ACPA does not explain how vimentin and fibrin β peptides are recognized. Cit-vimentin and fibrinβ peptide do not contain Cit-Gly, but instead have Ala-Cit motifs. Similarly, collagen II and enolase peptides also have the Ala-Cit motif. Although the vimentin peptide had restricted reactivity with affinity-purified IgG fractions, IgG purified on Cit-vimentin recognized all other peptides, even without Ala-Cit, such as EBNA-2 and filaggrin, indicating that antibodies specific for the Ala-Cit motif cross-react with Cit-Gly and thus Ala-Cit motif is also important in the recognition. Based on these, we designed an artificial peptide containing two Cit-Gly and two Ala-Cit motifs. This artificial peptide has a higher sensitivity to detect serum ACPA than fibrin β and filaggrin peptides, has a similar binding affinity, and can target in vitro the peptide-specific B cells of RA patients.

We determined the apparent binding affinities of Cit-peptides to affinity-purified antibodies by SPR biosensor analysis. The IgG fractions purified by affinity chromatography show a comparable *K*_D_ and a similar distribution of dissociation constants as RA serum antibodies. Most of the *K*_D_ values were within 1 micromolar–10 nanomolar and showed high heterogeneity. Since both the affinity-purified IgGs and the serum antibodies may contain the product of several B cell clones with the same specificity but different affinities, the obtained apparent *K*_D_ values are representing an average of these. While almost all serum samples bound to Cit-vimentin and to the multi-epitope peptide, only 30–60% of RA sera reacted with citrulline-containing collagen II, fibrin β and filaggrin19 peptides on the chip, although all samples were positive in ELISA. This may be explained by the difference between the static nature of the binding to ELISA plates in contrast to the dynamic flow rate over the surface of the chip, which induces a faster dissociation of the analyte from the peptide ligand-coated surfaces. Thus, antibodies with low affinity might be lost.

In concert with previous findings [39], we detected an inverse correlation between *K*_D_ values and the disease severity (DAS 28); in the case of Cit-filaggrin19 and Cit-fibrinβ peptides, the lowest *K*_D_ was associated with the most severe disease. A possible explanation could be that low binding affinity might allow higher degree of cross-reaction, leading to more extensive tissue damage.

ACPA play a pathological role in the development of RA, the perpetuation of inflammation, and the pathophysiology of the disease [46]. ACPA-positive patients have a faster progress of the disease and a worse radiological outcome [27,47], thus the detailed characterization of ACPA has high importance. The results presented here contribute to the understanding of the interactions between citrullinated epitopes and ACPA in RA patients, and thus may support the development of better diagnostics and precision therapy for RA.

## 4. Materials and Methods

### 4.1. Patients

We collected blood samples from RA patients (165 women and 45 men) diagnosed according to the revised classification criteria of American College of Rheumatology/European League Against Rheumatism (ACR/EULAR) [48]. The median age of patients was 63 years, and the median disease duration was 6 years. Blood samples were taken after the patients signed a written consent, and with ethical permission provided by the National Public Health and Medical Officer Service (49468-/2013/EKU (576/2013), 2 April 2014). Selected patients with a high level of anti-CCP antibodies were repeatedly recruited for the functional assays. A total of 120 age-matched healthy control sera were obtained from healthy volunteers.

### 4.2. ACPA Affinity Purification

We purified IgG from RA sera via Protein G agarose (Thermo Fischer Scientific, Waltham, MA, USA) affinity chromatography. To isolate ACPAs, Citrulline-containing peptides were covalently bound (5 mg/mL) to GE NHS HiTrap 1 mL column, according to the manufacturer’s instructions. Purified IgG was diluted 2× in 0.1 M NaH_2_PO_4_, 0.15 M NaCl, pH = 7 buffer and circulated through the column for 3 days. After washing, ACPA-containing fractions were eluted with 0.1 M glycine buffer (pH 2.5). The pH was immediately restored by adding 10 µL 2 M TRIS pH 9 to 1 mL eluted ACPA. The eluate fractions were dialyzed against PBS and after determining the IgG concentration via a Nanodrop Spectrophotometer (Thermo Scientific, Waltham, MA, USA), the samples were stored at −20 °C.

### 4.3. ELISA

ELISA was used to determine serum IgG ACPA concentrations and to test ACPA cross-reactions. The biotinylated, citrulline- or arginine-containing filaggrin19, vimentin, collagen II and the multi-epitope peptides were added at 1 µg/mL to the Medisorp plates (Greiner Bio-One GmbH, Kremsmünster, Germany) pre-coated with 5 µg/mL Neutravidin (Pierce Biotechnology, Rockford, IL, USA) overnight, at 4 °C, and then the plates were incubated for 1 h at 37 °C. EBNA2, α-enolase and fibrin β peptides were coated directly to Maxisorp (Nunc) plates at 2.5 µg/mL. After washing, the plates were blocked with 150 mM NaCl and 2% BSA containing PBS for 1 h, at 37 °C.

To determine the specific ACPA concentrations in sera a calibration curve was prepared from the affinity-purified ACPA as standard, in a two-fold dilution series, starting from 0.5 mg/mL. Serum samples were diluted 1:100. Dilution buffer was 2 M NaCl, 2% BSA in PBS. In cross-reaction assays, ACPAs were diluted to 0.1 mg/mL. Sera/ACPA samples were incubated overnight at 4 °C, shaking. After washing, the plates were incubated with 1:15,000 dilution of rabbit anti-human IgG HRP (H+L) (Southern biotech, Birmingham, AL, USA) for 1 h at 37 °C. Signal was developed with TMB substrate (Sigma-Aldrich, St. Louis, MO, USA) and the reaction was stopped with 2 N H_2_SO_4_. Plates were read at 450 nm with THERMO Multiscan EX ELISA-reader.

### 4.4. SPR Analysis

All experiments were conducted using a ProteOn™ XPR36 Protein Interaction Array System from Bio-Rad (Bio-Rad, Hercules, CA, USA). All solutions and sensor chips were purchased from Bio-Rad. Citrulline-containing peptides were immobilized on GLH sensor chip, with amine coupling strategy. Immobilization buffers were selected separately for each peptide, according to the isoelectric point of the peptide. Running buffer was 0.005% PBST pH 7.4.

For immobilization, the sensor chip surface was activated with a 1:1 mixture of 400 mM EDAC and 100 mM Sulfo-NHS for 300 s at a flow rate of 30 µL/min. Immobilization buffers were selected separately for each peptide using the pH scouting procedure, using the following buffers: sodium acetate buffer (10 mmol/L, pH 4, 4.5, 5.0), phosphate buffer (10 mmol/L, pH 6, 6.5, 7, 7.5 and 8) and borate buffer (10 mmol/L, pH 9 and 9.5). Peptides were solubilized in each buffer at a final concentration of 10 µg/mL. The selected buffer and immobilized peptide quantities are reported in Table 3. Each peptide, solubilized in the previously selected immobilization buffer (10 µg/mL), was injected for 300 s at a flow rate of 30 µL/min. To deactivate free reactive sites, ethanolamine-HCl (1 mol/L, pH 8.5) was injected for 300 s at a flow rate of 30 µL/min. The reference channel was activated by injecting NHS/EDC (1:1) and PBST and deactivated with ethanolamine–HCl.

Peptides that were not able to bind to the surface of the GLH chip were biotinylated and then coupled to neutravidin-functionalized NLC sensor chips (Bio-Rad). After the immobilization of the biotinylated peptides (2.5 µg/mL, 30 µL/min, 300 s), the excess of neutravidin binding sites were neutralized by biocytin (Sigma) in a concentration of 10 µg/mL for 200 s at a flow rate of 30 µL/min.

Affinity-purified anti-citrullinated peptide antibodies were diluted in running buffer to final concentrations of 2000, 1000, 500, 250, and 125 nmol/L. Serum samples were used in two-fold dilutions, starting dilution was 10× in running buffer. Diluted samples were injected over each immobilized peptide for 120 s contact time at a flow rate of 50 µL/min. Dissociation was performed over a 600 s at a flow rate of 50 µL/min, and finally the chip surface was regenerated by injecting a glycine solution (10 mmol/L, pH 2) for 30 s, 100 µL/min and washed with running buffer at 90 µL/min flow rate for 150 s. Binding kinetics and data analysis were calculated with Bio-Rad ProteOn Manager software, Langmuir kinetic model [41].

### 4.5. Peptide Synthesis

The peptides were synthesized manually using conventional solid-phase peptide synthesis method according to Fmoc/^t^Bu strategy as described previously [31,37,43] (Table 1). The N-terminus of some peptides were acetylated and the C-terminus of the analogs were always amidated. The N-terminus of fibrin and multi-epitope peptide or C-terminus of filaggrin 19, vimentin and collagen II were biotinylated using biotinyl-6-amino-hexanoic acid. In the case of multi-epitope 4,7,10-trioxa-1,13-tridecanediamino succinic acid linker (Ttds) was used [37]. All peptides and bioconjugates were characterized by analytical RP-HPLC and ESI mass spectrometry.

### 4.6. Preparation and Characterization of Bifunctional PLGA NPs

Carboxylate-functionalized PLGA NPs were prepared by the nanoprecipitation method as described previously [32]. The average diameter of NPs was 160–180 nm determined by dynamic light scattering (DLS). The chain end carboxylic groups of PLGA are available at the surface of NPs in a quantity of 4000–5000/NP. Those were converted to active-ester using *N*-hydroxysuccinimide and 1-ethyl-3-(3-dimethylaminopropyl)-carbodiimide in aqueous environment. Targeting and complement-activating peptides were chemically coupled to the active ester groups on the PLGA NPs. The application of peptide mixture (1:1 molar ratio) resulted in their uniform distribution on the NP’s surface.

### 4.7. Detection of Cit-Peptide-Specific Antibody Producing Cells

Mononuclear cells (PBMC) were prepared from the blood of RA patients and healthy volunteers as described previously [38]. PBMC (10^6^/mL) in RPMI-1640/10% FCS were stimulated with 1 μg/mL R848 polyclonal activator, 10 ng/mL recombinant IL-2, 50 ng/mL IL-21 and 1 μg/mL CD40 ligand (CD40L). At day three cells were harvested, counted and treated at 1:1000 with bifunctional PLGA NP covered with the multi-epitope and the modified complement-activating peptides for 1 h. Unbound NPs were washed and 1% pooled normal human serum (NHS) or heat inactivated NHS as a control were added at 37 °C for 30 min. The cells were washed again and 4 × 10^5^ PBMCs/well were transferred to ELISpot plates pre-coated with the citrulline-containing multi-epitope peptide and with anti-IgG, respectively. The spots were developed after 18 h according to the manufacturer’s instruction (Mabtech, Stockholm, Sweden). The frequency of Cit-containing multi-epitope peptide-specific IgG and non-specific IgG-producing cells was determined using a C.T.L. Immunospot analyzer (CTL-Europe GmbH, Bonn, Germany).

### 4.8. Statistical Analysis

The data were statistically analyzed by the two-tailed *t*-test and Pearson correlation coefficients were calculated. We analyzed the results with GRAPHPAD PRISM software (GraphPad Prism 5 Software, La Jolla, CA, USA). *p* < 0.05 was considered significant.

## Figures and Tables

**Table 1:** List of citrulline containing peptides used in the assays.

Peptides	Sequences	References
Filaggrin (306–326)	Ac-SHQEST**X**G**X**SXGRSGRSGSK-NH_2_	[33]
Collagen II (359–369)	Ac-A**X**GLTG**X**PGDA-NH_2_	[7]
Fibrin β (60–74)	H-**X**PAPPPISGGGY**X**A**X**-NH_2_	[8,34]
Vimentin (65–77)	Ac-SAVRA**X**SSVPGVRK-NH_2_	[10]
α-enolase (5–21)	Ac-KIHA**X**EIFDS**X**GNPTVE-NH_2_	[35]
EBNA-2 (341–361)	Ac-GQS**X**GQS**X**G**X**G**X**G**X**G**X**G**X**GKG-NH_2_	[36]
multi-epitope	H-Ttds-A**X**A**X**GSGSG**X**G**X**G-NH_2_	

**X** stands for citrulline; Ttds, linker 1,13-diamino-4,7,10-trioxatridecan-succinamic acid. For screening the serum samples by ELISA both citrulline and arginine containing variants of the same peptides were used.

**Table 2:** *K*_D_ of affinity purified IgG of anti-peptide antibodies as measured by SPR (surface plasmon resonance).

Peptides	Filaggrin	Collagen II	Fibrin β	Vimentin	Multi-Epitope
filaggrin19 ACPA	4.05 × 10^−7^	2.10 × 10^−6^	1.65 × 10^−6^	nb *	1.33 × 10^−6^
collagen II ACPA	5.32 × 10^−8^	6.10 × 10^−8^	8.44 × 10^−8^	1.31 × 10^−7^	7.17 × 10^−8^
fibrin β ACPA	3.56 × 10^−7^	1.21 × 10^−6^	8.34 × 10^−7^	3.86 × 10^−7^	2.75 × 10^−7^
vimentin ACPA	5.58 × 10^−7^	nb	1.65 × 10^−6^	nb	nb
α-enolase ACPA	nb	8.96 × 10^−8^	2.47 × 10^−8^	nb	2.94 × 10^−8^

* nb: no binding.

**Table 3:** Characteristics of peptide immobilization on GLH chip.

Peptide	Immobilization Buffer	Immobilized Peptide (RU)
Filaggrin (306–326)	sodium acetate pH 4	500
Vimentin (65–77)	borate pH 9	5700
Multi-epitope	borate pH 9.5	3100

## Abbreviations

ACPA	Anti-Citrullinated Protein/peptide Antibodies
Cit	Citrulline
DAS	Disease Activity Score
EBNA	Epstein-Barr virus Nuclear Antigen
EBV	Epstein-Barr virus
IVIG	Intravenous Immunoglobulin
NET	Neutrophil Extracellular Trap
NHS	Normal Human Sera
NP	Nanoparticles
PBMC	Peripheral Blood Mononuclear Cells
PLGA	Poly(lactic-co-glycolic acid)
RA	Rheumatoid arthritis
SPR	Surface Plasmon Resonance
